# Supplementary material for: Outcome After Laparoscopic Compared to Open Interval Debulking Surgery for Advanced Stage Ovarian Cancer: A Systematic Review and Meta-Analysis
Source: Cancers (Basel). 2025 Nov 30;17(23):3858. doi: 10.3390/cancers17233858 (PMC12691450; doi:10.3390/cancers17233858)
Supplement: Supplementary file 1 [file cancers-17-03858-s001.zip › Supplementary Figures.pdf]

Supplementary Figures

Study or Subgroup	MIS Events	MIS Total	LPT Events	LPT Total	Weight	Risk ratio IV, Random, 95% CI
Abitbol et al.	57	57	20	22	7.6%	1.11 [0.96 , 1.29]
Brown et al.	51	53	86	104	10.9%	1.16 [1.05 , 1.29]
Brown J et al.	113	122	246	293	14.2%	1.10 [1.03 , 1.18]
Davidson BA et al.	26	26	202	245	13.5%	1.19 [1.10 , 1.29]
Favero et al.	10	10	11	11	6.0%	1.00 [0.84 , 1.19]
Gueli Alletti et al.	30	30	65	65	16.5%	1.00 [0.95 , 1.05]
Melamed et al.	220	277	1234	1595	14.8%	1.03 [0.96 , 1.10]
Persenaire et al.	593	882	2937	4414	16.4%	1.01 [0.96 , 1.06]
**Total (HKSJ[a])**		**1457**		**6749**	**100.0%**	**1.07 [1.01 , 1.14]**
Total events:	1100		4801			

Test for overall effect: T = 2.71, df = 7 (P = 0.03)

Heterogeneity: Tau² (REML[b]) = 0.00; Chi² = 23.25, df = 7 (P = 0.002); I² = 71%

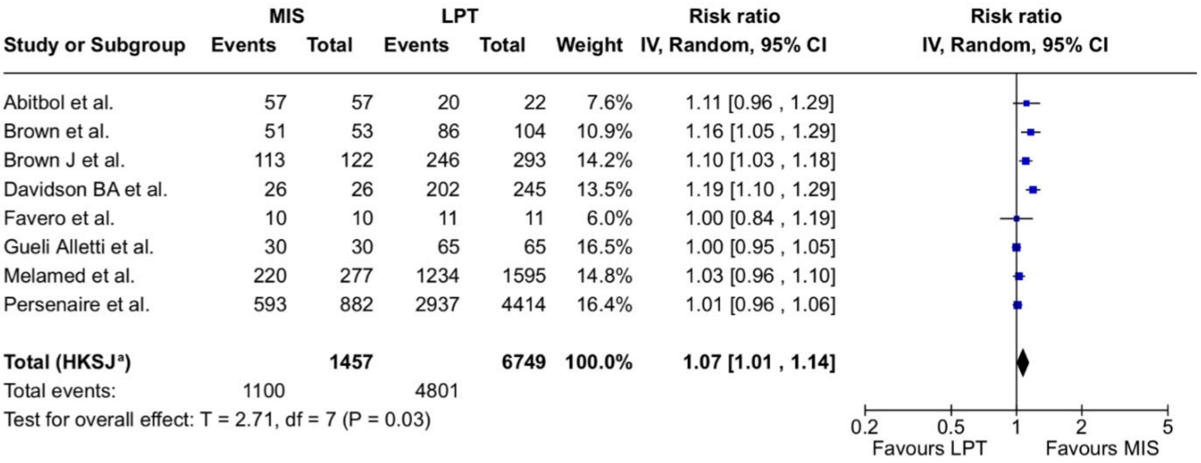

Footnotes

[a]CI calculated by Hartung-Knapp-Sidik-Jonkman method.

[b]Tau² calculated by Restricted Maximum-Likelihood method.

Supplementary Figure S1. Rate of R0/R1 (optimal) resection after minimally invasive compared to open interval debulking surgery. Abbreviations: R0/R1, optimal resection to residual disease <10 mm, MIS, minimally invasive surgery; LPT, laparotomy; 95% CI, confidence interval; HKSJ, CI calculated by Hartung-Knapp-Sidik-Jonkman method, REML, Tau2 calculated by Restricted Maximum-Likelihood method.

A. R0 resection; subgroup analysis

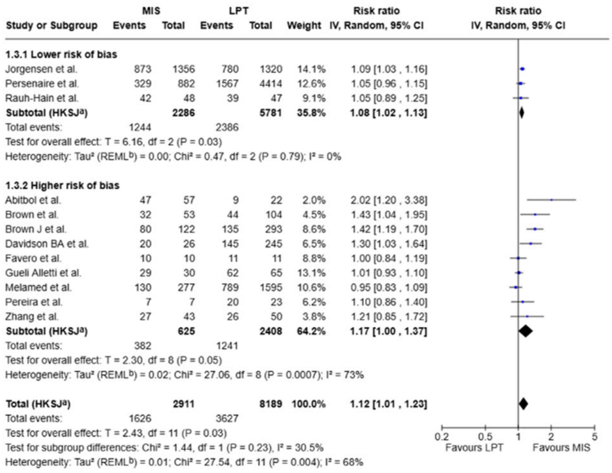

(a) Overall risk of bias (lower vs. higher)

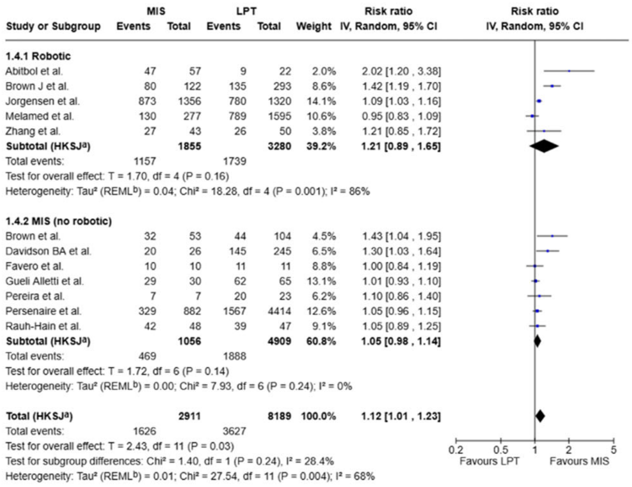

(b) Surgical modality (robotic vs. MIS (no robotic))

B. Overall survival; subgroup analysis

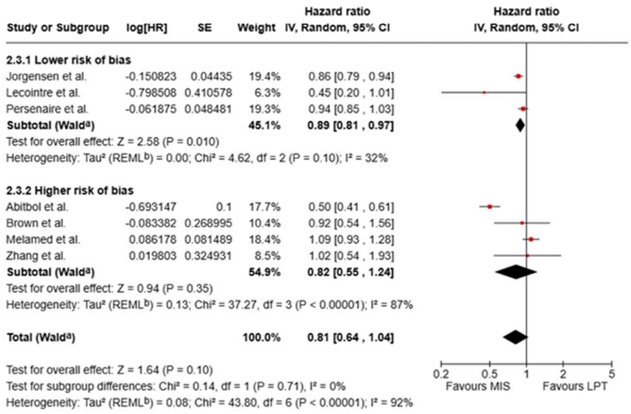

(a) Overall risk of bias (lower vs. higher)

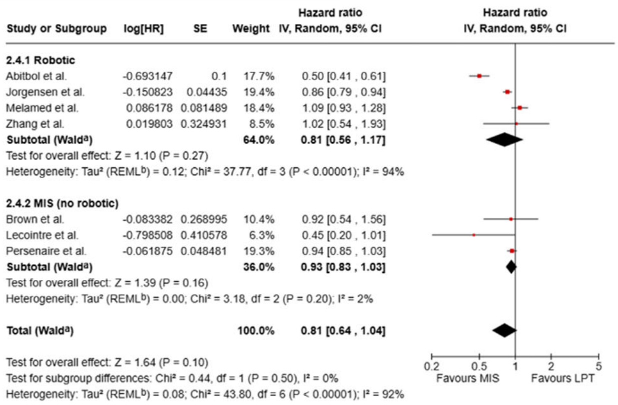

(b) Surgical modality (robotic vs. MIS (no robotic))

C. Progression- free survival; subgroup analysis

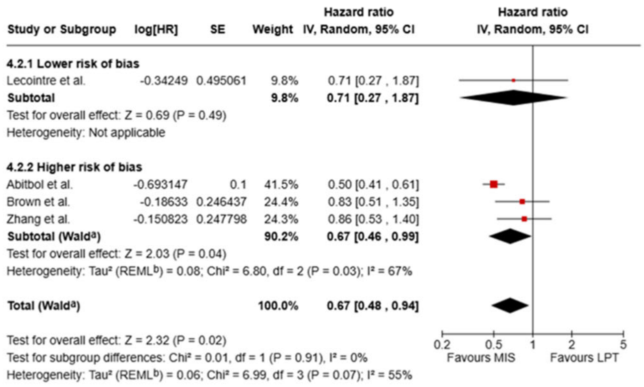

(a) Overall risk of bias (lower vs. higher)

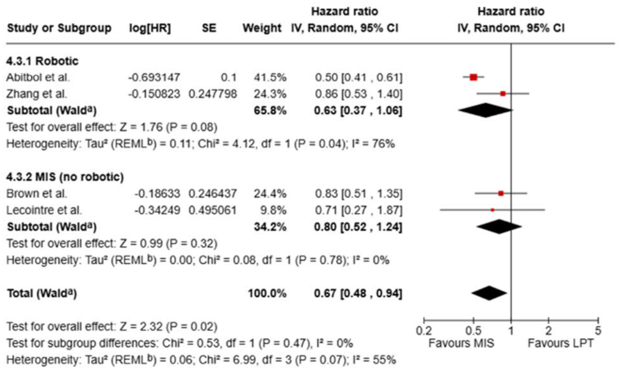

(b) Surgical modality (robotic vs. MIS (no robotic))

Supplementary Figure S2. Subgroup analyses by overall risk of bias (a) and surgical modality in the minimally invasive surgery cohort (b), labelled as robotic and MIS (no robotic). A. Subgroup analysis for complete cytoreduction (R0 resection). B. Overall survival per subgroup. C. Subgroup analysis for progression-free survival. MIS, minimally invasive surgery; LPT, laparotomy; HR, hazard ratio; SE, standard error; IV, inverse variance; Wald, Wald-type method.

	MIS		LPT			Risk ratio	Risk ratio
Study or Subgroup	Events	Total	Events	Total	Weight	IV, Random, 95% CI	IV, Random, 95% CI
Abitbol et al.	43	57	19	22	31.0%	0.87 [0.70 , 1.09]	
Brown J et al.	73	122	205	293	57.3%	0.86 [0.73 , 1.01]	
Favero et al.	8	10	10	11	11.7%	0.88 [0.61 , 1.26]	
**Total (HKSJ[a])**		**189**		**326**	**100.0%**	**0.86 [0.83 , 0.89]**	
Total events:	124		234				

Test for overall effect: T = 17.75, df = 2 (P = 0.003)

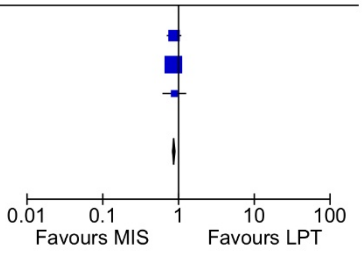

Heterogeneity: Tau² (REML[b]) = 0.00; Chi² = 0.03, df = 2 (P = 0.98); I² = 0%

Supplementary Figure S3. Rate of recurrence after minimally invasive compared to open interval debulking surgery. Abbreviations: MIS, minimally invasive surgery; LPT, laparotomy; 95% CI, confidence interval; HKSJ, CI calculated by Hartung-Knapp-Sidik-Jonkman method, REML, Tau2 calculated by Restricted Maximum-Likelihood method.

Odds ratio | Risk ratio | Risk difference | Fixed effect | Random effects | DL | REML | Wald | HKSJ

Scale 5 Download as SVG

Study or Subgroup	MIS Events	MIS Total	LPT Events	LPT Total	Weight	Risk ratio IV, Random, 95% CI
✓ Abitbol et al.	47	57	9	22	2.0%	2.02 [1.20 , 3.38]
✓ Brown et al.	32	53	44	104	4.5%	1.43 [1.04 , 1.95]
✓ Brown J et al.	80	122	135	293	8.6%	1.42 [1.19 , 1.70]
✓ Davidson BA et al.	20	26	145	245	6.5%	1.30 [1.03 , 1.64]
✓ Favero et al.	10	10	11	11	8.8%	1.00 [0.84 , 1.19]
✓ Gueli Alletti et al.	29	30	62	65	13.1%	1.01 [0.93 , 1.10]
✓ Jorgensen et al.	873	1356	780	1320	14.1%	1.09 [1.03 , 1.16]
✓ Melamed et al.	130	277	789	1595	10.6%	0.95 [0.83 , 1.09]
✓ Pereira et al.	7	7	20	23	6.2%	1.10 [0.86 , 1.40]
✓ Persenaire et al.	329	882	1567	4414	12.6%	1.05 [0.96 , 1.15]
✓ Rauh-Hain et al.	42	48	39	47	9.1%	1.05 [0.89 , 1.25]
✓ Zhang et al.	27	43	26	50	3.8%	1.21 [0.85 , 1.72]
**Total (HKSJ[a])**		**2911**		**8189**	**100.0%**	**1.12 [1.01 , 1.23]**
Total events:	1626		3627			

Test for overall effect: T = 2.43, df = 11 (P = 0.03)

Heterogeneity: Tau² (REML[b]) = 0.01; Chi² = 27.54, df = 11 (P = 0.004); I² = 68%

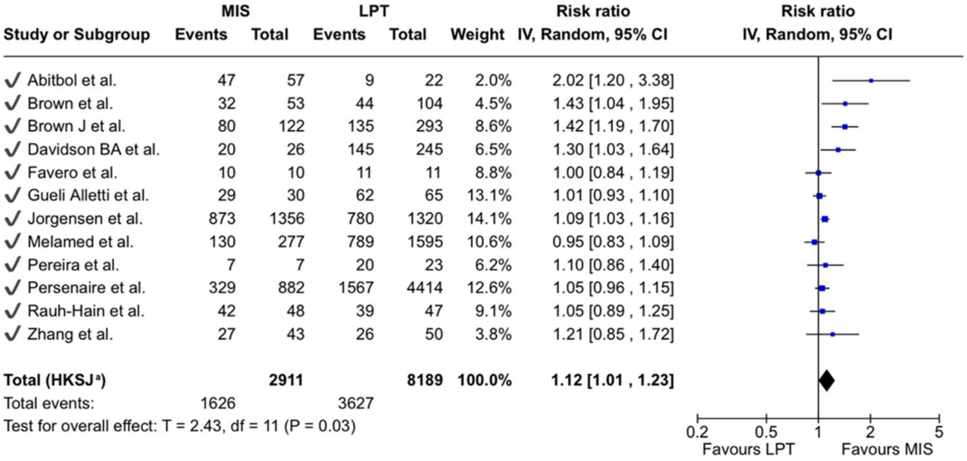

Odds ratio | Risk ratio | Risk difference | Fixed effect | Random effects

Scale 5 Download as SVG

Study or Subgroup	MIS Events	MIS Total	LPT Events	LPT Total	Weight	Risk ratio IV, Fixed, 95% CI
✓ Abitbol et al.	47	57	9	22	0.5%	2.02 [1.20 , 3.38]
✓ Brown et al.	32	53	44	104	1.4%	1.43 [1.04 , 1.95]
✓ Brown J et al.	80	122	135	293	4.2%	1.42 [1.19 , 1.70]
✓ Davidson BA et al.	20	26	145	245	2.4%	1.30 [1.03 , 1.64]
✓ Favero et al.	10	10	11	11	4.4%	1.00 [0.84 , 1.19]
✓ Gueli Alletti et al.	29	30	62	65	18.6%	1.01 [0.93 , 1.10]
✓ Jorgensen et al.	873	1356	780	1320	37.7%	1.09 [1.03 , 1.16]
✓ Melamed et al.	130	277	789	1595	7.4%	0.95 [0.83 , 1.09]
✓ Pereira et al.	7	7	20	23	2.3%	1.10 [0.86 , 1.40]
✓ Persenaire et al.	329	882	1567	4414	15.2%	1.05 [0.96 , 1.15]
✓ Rauh-Hain et al.	42	48	39	47	4.8%	1.05 [0.89 , 1.25]
✓ Zhang et al.	27	43	26	50	1.1%	1.21 [0.85 , 1.72]
**Total**		**2911**		**8189**	**100.0%**	**1.08 [1.04 , 1.12]**
Total events:	1626		3627			

Test for overall effect: T = 0.00, df = 11 (P < 0.00001)

Heterogeneity: Chi² = 27.54, df = 11 (P = 0.004); I² = 60%

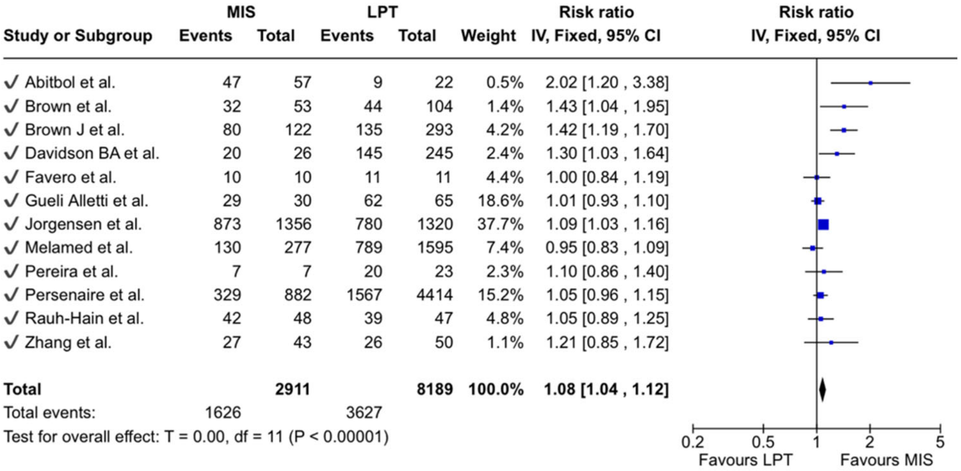

Supplementary Figure S4: Example for Sensitivity Analysis comparing R0 resection in a random-effects model (A) with a fixed-effect model with inverse variance (B). MIS, minimally invasive surgery; LPT, laparotomy; IV, inverse variance.
