# Supplementary material for: Outcome After Laparoscopic Compared to Open Interval Debulking Surgery for Advanced Stage Ovarian Cancer: A Systematic Review and Meta-Analysis
Source: Cancers (Basel). 2025 Nov 30;17(23):3858. doi: 10.3390/cancers17233858 (PMC12691450; doi:10.3390/cancers17233858)
Supplement: Supplementary file 1 [file cancers-17-03858-s001.zip › Supplementary Tables.pdf]

Supplementary Table S1. Study characteristics in detail.

Author	Year	Age, years		P-value	Chemotherapy cycles, n (median/mean, range/SD)		P-value	Follow-up, months (median, range)		P-value	Primary outcomes	Secondary outcomes
		LPT	MIS		LPT	MIS		LPT	MIS			
**Abitbol**	2019	Mean: 65.4 ±9.2	NR	NR	NR	NR	NR	Median: 36.2 (9-104.3)	Median: 41 (6-86)	NR	OS, PFS	Perioperative Outcomes
**Brown**	2019	Mean: 67.1 ± 9.6	Mean: 66.6 ± 11	0.79	Median: 3 (1-7)	Median: 3 (2-6)	0.67	NR	NR	NR	OS, PFS	Perioperative Outcomes
**Brown J**	2021	NR	NR	NR	NR	NR	NR	NR	NR	NR	OS, PFS	Perioperative Outcomes, recurrence
**Davidson BA**	2018	Median: 63.9 (34.1-84.8)	Median: 63.9 (34.1-84.8)	-	Median: 4 (2-10)	Median: 4 (2-10)	NR	NR	NR	NR	DSS	Perioperative outcomes, surgical complexity scores (SCS)
**Favero**	2015	Mean: 61.3 (41-80)	Mean: 58.3 (42-73)	0.47	Mean/median: 6	Mean/median: 6	NR	Median: 36 (24-48)	Median: 20 (12-26)	0.02	Feasibility and operative outcomes	OS, chemotherapy free interval
**Gueli Alletti**	2016	Median: 59 (48-80)	Median: 62 (40-81)	0.78	NR	NR	NR	Median: 28	Median: 28	0.786	PFS	Perioperative outcomes, evaluation of quality of life with the General Well-Being Schedule (GWBS)
**Jorgensen**	2023	Mean: 63.8	Mean: 65.2	<0.001	NR	NR	NR	Median: 28	Median: 28	NR	OS	5-year survival, 30- and 90-day postoperative mortality, perioperative outcomes
**Lecointre**	2022	Mean: 61.7 ± 9.5	Mean: 62.8 ± 11.2	0.74	Mean: 4.4 ± 1.1	Mean: 5.1 ± 1.2	0.009	Median: 24.9	Median: 24.9	NR	OS, PFS	Intra- and postoperative morbidity
**Melamed**	2017	Mean: 63.9 ± 11.7	Mean: 63.2 ± 11.1	0.12	NR	NR	NR	Median: 32	Median: 32	NR	OS	3-year survival, perioperative outcomes
**Pereira**	2022	NR	NR	NR	Mean/median: 3	Mean/median: 3	-	Median: 31 (0.5-116)	Median: 31 (0.5-116)	NR	OS, PFS	Perioperative outcomes, 30-day mortality
**Persenaire**	2022	NR	NR	NR	NR	NR	NR	NR	NR	NR	OS	Sociodemographic and clinical variables associated with surgical approach
**Pomel**	2021	Median: 70 (66-76)	Median: 72.5 (45-89)	NR	Median: 4 (3-6)	Median: 5.5 (3-7)	NR	NR	Median: 26	NR	Survival status	Perioperative outcomes, oncological safety, quality of life

**Rauh-Hain**	2024	Median: 63 (57-70)	Median: 61 (55-68)	NR	NR	NR	NR	NR	NR	NR	lead-in pilot phase, asses the feasibility of an RCT; rate of laparoconversion, complete gross resection rates,	Perioperative outcomes, health-related quality of life
**Zhang**	2021	Median: 63	Median: 66.2	0.1	Mean: 4	Mean: 4.4	0.1	Median: 27	Median: 31.8	0.49	OS, PFS, surgical morbidity,recurrence	Perioperative outcomes

Abbreviations: MIS, minimally invasive surgery; LPT, laparotomy; n, number of patients; SD, standard deviation; NR, not reported.

Supplementary Table S2. Rate of intraoperative complications and blood loss in minimally invasive compared to open interval debulking surgery.

Author	Year	Intraoperative complications		P-value	Blood loss, mL (median/mean, range/SD)		P-value
		LPT, n (%)	MIS, n (%)		LPT	MIS	
**Abitbol**	2019	NR	NR	NR	Mean: 135 ± 210, Median: 100 (10-1250)	Mean: 505 ± 599	NR
**Brown**	2019	3 (2.9)	1 (1.9)	NR	Mean: 156 ± 151	Mean: 278 ± 277	<0.001
**Brown J**	2021	NR	NR	NR	Mean: 181.5	Mean: 326.2	<0.001
**Davidson BA**	2018	23 (9.4)	0 (0)	NR	NR	NR	NR
**Favero**	2015	NR	0 (0)	NR	Mean: 180 (90-320)	NR	NR
**Gueli Alletti**	2016	NR	NR	NR	Median: 100 (50-200)	Median: 200 (100-400)	0.047
**Jorgensen**	2023	NR	NR	NR	NR	NR	NR
**Lecointre**	2022	5 (12.5)	6 (16.2)	0.89	Mean: 376.9 ± 411.7	Mean: 613 ± 434.7	0.03
**Melamed**	2017	NR	NR	NR	NR	NR	NR
**Pereira**	2022	1 (4.3)	0 (0)	0.57	NR	NR	NR
**Persenaire**	2022	NR	NR	NR	NR	NR	NR
**Pomel**	2021	NR	2 (6.25)	NR	Median: 150 (0-500)	NR	NR
**Rauh-Hain**	2024	3 (6)	3 (6)	NR	Median: 100 (50-250)	Median: 200 (100-400)	NR
**Zhang**	2021	NR	NR	NR	Mean: 98.7	Mean: 372.9	<0.001

Abbreviations: MIS, minimally invasive surgery; LPT, laparotomy; mL, millilitre; SD, standard deviation; n, number of patients; NR, not reported.

Supplementary Table S3. Duration to initiation of adjuvant chemotherapy, readmission rate and recurrence rate after minimally invasive compared to open interval debulking surgery.

Author	Year	Days to adjuvant chemotherapy, days (median, range/SD)		P-value	Readmission		P-value	Recurrence		P-value
		LPT	MIS		LPT, n (%)	MIS, n (%)		LPT, n (%)	MIS, n (%)	
**Abitbol**	2019	Median: 13 (6-75)	NR	NR	NR	NR	NR	19 (86.4)	43 (75.4)	NR
**Brown**	2019	NR	NR	NR	NR	NR	NR	NR	NR	NR
**Brown J**	2021	NR	NR	NR	29 (9.9)	7 (5.7)	0.2	205 (70)	73 (59.8)	0.06
**Davidson BA**	2018	NR	NR	NR	NR	2 (7.4)	NR	NR	NR	NR
**Favero**	2015	NR	NR	NR	NR	NR	NR	10 (90.9)	8 (80)	NR
**Gueli Alletti**	2016	Median: 20 (10-30)	Median: 35 (19-60)	0.003	NR	NR	NR	NR	NR	NR
**Jorgensen**	2023	NR	NR	NR	63 (3.1)	55 (2.7)	0.39	NR	NR	NR
**Lecointre**	2022	Median: 31.6 ± 17.4	Median: 30.7 ± 24.4	0.71	5 (12.5)	1 (2.7)	0.2	NR	NR	NR
**Melamed**	2017	NR	NR	NR	97 (3.7)	24 (5.3)	0.26	NR	NR	NR
**Pereira**	2022	NR	NR	NR	NR	NR	NR	NR	NR	NR
**Persenaire**	2022	NR	NR	NR	41 (3)	198 (3)	0.99	NR	NR	NR
**Pomel**	2021	Median: 36.8	NR	NR	NR	2 (6.25)	NR	NR	NR	NR
**Rauh-Hain**	2024	NR	NR	NR	NR	NR	NR	NR	NR	NR
**Zhang**	2021	Mean: 31.4	Mean: 32.5	0.7	8 (16)	3 (7.9)	NR	NR	NR	NR

Abbreviations: MIS, minimally invasive surgery; LPT, laparotomy; SD, standard deviation; n, number of patients; NR, not reported.

Supplementary Table S4. Risk of bias assessment (ROBINS-I / RoB2).

**Author**	**Year**	**Design**	**Confounding**	**Selection**	**Classification**	**Deviations**	**Missing data**	**Outcome measurement**	**Selective reporting**	**Overall ROBINS-I**
Abitbol	2019	Retrospective	Serious	Moderate	Low	Moderate	Moderate	Low	Low	**Serious**
Brown	2019	Retrospective	Serious	Moderate	Low	Moderate	Moderate	Moderate	Low	**Serious**
Brown J	2021	Retrospective	Serious	Moderate	Low	Moderate	Moderate	Moderate	Low	**Serious**
Davidson	2018	Retro-/prospective	Serious	Serious	Low	Moderate	Moderate	Moderate	Low	**Serious**
Favero	2015	Prospective	Serious	Serious	Low	Moderate	Moderate	Moderate	Low	**Serious**
Gueli Alletti	2016	Retrospective	Serious	Moderate	Low	Moderate	Moderate	Low	Low	**Serious**
Jørgensen	2023	Retrospective	Moderate	Low	Low	Moderate	Moderate	Moderate	Low	**Moderate**
Lecointre	2022	Retrospective	Moderate	Moderate	Low	Moderate	Low	Low	Moderate	**Moderate**
Melamed	2017	Retrospective	Moderate	Moderate	Low	Moderate	Low	Moderate	Low	**Moderate**
Pereira	2022	Retrospective	Serious	Serious	Low	Moderate	Moderate	Moderate	Low	**Serious**
Persenaire	2022	Retrospective	Moderate	Serious	Low	Moderate	Moderate	Low	Moderate	**Moderate**
Pomel	2021	Prospective	Serious	Moderate	Low	Moderate	Moderate	Low	Low	**Serious**
Zhang	2021	Retrospective	Serious	Serious	Low	Moderate	Moderate	Moderate	Low	**Serious**
Rauh-Hain	2024	Randomized controlled trial	Assessed with RoB 2	–	–	–	–	–	–	**Low risk of bias**

Supplementary Table S5. Certainty of evidence according to the Guideline Development Tool platform (GradePro).

MIS compared to LPT for Interval debulking surgery

Patient or population: Interval debulking surgery

Setting:

Intervention: MIS

Comparison: LPT

Outcomes	Anticipated absolute effects* (95% CI)		Relative effect (95% CI)	№ of participants (studies)	Certainty of the evidence (GRADE)	Comments
	Risk with LPT	Risk with MIS				
R0 resection	443 per 1'000	**496 per 1'000** (447 to 545)	**RR 1.12** (1.01 to 1.23)	11100 (12 non-randomised studies)	⊕⊕⊕⊕ High	
R0/R1 resection	711 per 1'000	**761 per 1'000** (718 to 811)	**RR 1.07** (1.01 to 1.14)	8206 (8 non-randomised studies)	⊕⊕⊕◯ Moderate	
Overall survival	0 per 1'000	**NaN per 1'000** (-- to --)	**HR 0.81** (0.60 to 1.10)	(7 non-randomised studies)	⊕◯◯◯ Very low	
Progression-free survival	0 per 1'000	**NaN per 1'000** (-- to --)	**HR 0.67** (0.42 to 1.06)	(4 non-randomised studies)	⊕⊕◯◯ Low	
Blood loss	-	SMD **0.58 lower** (0.82 lower to 0.35 lower)	-	508 (5 non-randomised studies)	⊕⊕◯◯ Low	
Operative time	-	SMD **0.5 higher** (0.11 higher to 0.89 higher)	-	459 (6 non-randomised studies)	⊕⊕◯◯ Low	
Length of stay	-	SMD **0.79 lower** (1.06 lower to 0.52 lower)	-	15757 (10 non-randomised studies)	⊕⊕⊕◯ Moderate	

Supplementary Table S5. Certainty of evidence according to the Guideline Development Tool platform (GradePro).

MIS compared to LPT for Interval debulking surgery

Patient or population: Interval debulking surgery

Setting:

Intervention: MIS

Comparison: LPT

Outcomes	Anticipated absolute effects* (95% CI)		Relative effect (95% CI)	№ of participants (studies)	Certainty of the evidence (GRADE)	Comments
	**Risk with LPT**	**Risk with MIS**				
Intraoperative complications	76 per 1'000	**73 per 1'000** (36 to 150)	**RR 0.96** (0.47 to 1.98)	636 (5 non-randomised studies)	⨁⨁◯◯ Low	
Postoperative complications	386 per 1'000	**193 per 1'000** (127 to 294)	**RR 0.50** (0.33 to 0.76)	988 (8 non-randomised studies)	⨁⨁⨁◯ Moderate	
Days to adjuvant chemotherapy	-	SMD **0.26 lower** (4.09 lower to 3.57 higher)	-	172 (2 non-randomised studies)	⨁◯◯◯ Very low	
Readmission	40 per 1'000	**33 per 1'000** (16 to 66)	**RR 0.82** (0.41 to 1.64)	7698 (5 non-randomised studies)	⨁◯◯◯ Very low	
Recurrence	718 per 1'000	**617 per 1'000** (596 to 639)	**RR 0.86** (0.83 to 0.89)	515 (3 non-randomised studies)	⨁◯◯◯ Very low	

***The risk in the intervention group** (and its 95% confidence interval) is based on the assumed risk in the comparison group and the **relative effect** of the intervention (and its 95% CI).

CI: confidence interval; **HR:** hazard ratio; **RR:** risk ratio; **SMD:** standardised mean difference

GRADE Working Group grades of evidence
High certainty: we are very confident that the true effect lies close to that of the estimate of the effect.
Moderate certainty: we are moderately confident in the effect estimate: the true effect is likely to be close to the estimate of the effect, but there is a possibility that it is substantially different.
Low certainty: our confidence in the effect estimate is limited: the true effect may be substantially different from the estimate of the effect.
Very low certainty: we have very little confidence in the effect estimate: the true effect is likely to be substantially different from the estimate of effect.
